# Melatonin mitigates autophagy: unlocking conditional resilience in sheep trophoblast cells exposed to a hypoxic environment

**DOI:** 10.1530/RAF-25-0084

**Published:** 2025-12-02

**Authors:** Irene Viola, Paolo Accornero, Elisa Quarati, Isabella Manenti, Silvia Miretti, Francisco Canto, José Alfonso Abecia, Paola Toschi

**Affiliations:** ^1^ Department of Veterinary Sciences, University of Turin, Torino, Italy; ^2^ Centro Regional de Investigación Remehue, Instituto de Investigaciones Agropecuarias, Osorno, Región de Los Lagos, Chile; ^3^ Instituto Universitario de Investigación en Ciencias Ambientales (IUCA), University of Zaragoza, Zaragoza, Spain

**Keywords:** sheep, placenta, melatonin, hypoxia stress, autophagy

## Abstract

**Graphical Abstract:**

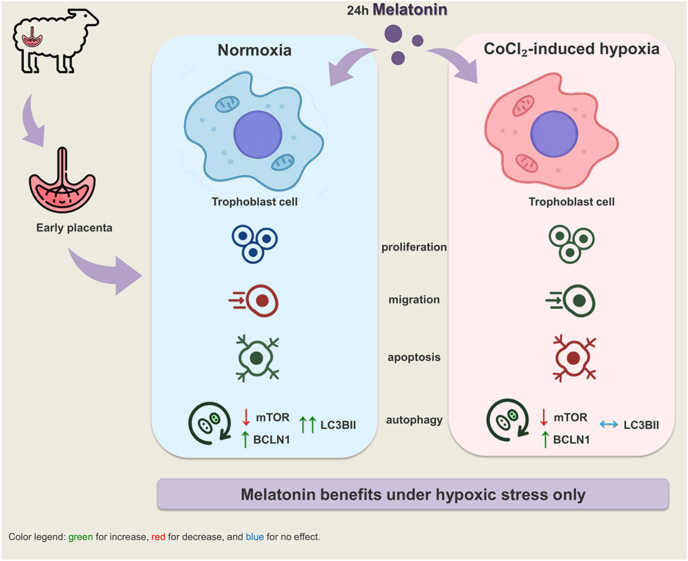

**Abstract:**

Melatonin is a key molecule in supporting pregnancy success in sheep, particularly under suboptimal conditions. In humans, melatonin is also known for its antioxidant properties. In addition, it has recently been reported that melatonin differentially drives cell fate in normal vs altered trophoblast cells. Given that, we hypothesize that melatonin is a potential partner for trophoblasts to overcome a hypoxic environment during the early stage of pregnancy. Here, we explore the effect of melatonin on early trophoblast cell behavior and its potential mitigating effect in CoCl_2_-induced hypoxia. Cell functionality and autophagy modulation were studied on ovine primary trophoblast cells (oTCs) 24 h treated with 250 µM melatonin with/without 200 µM CoCl_2_. First, melatonin exerts its antioxidant effects by reducing H_2_O_2_ levels under hypoxic cellular conditions (*P* < 0.0001). CoCl_2_ suppressed cell proliferation and migration (*P* < 0.0001); however, melatonin supplementation partially restored oTCs functionality (*P* < 0.05). Melatonin-mediated cytoprotective effects are manifested even through the modulation of cell fate mechanisms, particularly autophagy and apoptosis. Increased protein expression of autophagic markers (BCLN1 and LC3BII/LC3BI ratio) in concomitance with a decreased phosphorylation of mTOR was observed in CoCl_2_-treated cells (*P* < 0.01), while a reduced rate of autophagy was detected following melatonin co-treatment (*P* < 0.01). Similarly, melatonin attenuates the CoCl_2_-induced increase in apoptosis when administered concurrently (5.5 vs 1.8%, *P* < 0.01). These findings suggest that melatonin promotes autophagy over apoptosis, indicating a shift toward cell survival mechanisms. In addition, melatonin enhances cell functionality under hypoxia, suggesting the conceptus benefits from melatonin, particularly when it is forced to grow in a suboptimal environment.

**Lay summary:**

Melatonin is known for its role in regulating reproduction in sheep; however, very little is known about the role it plays in the development of the placenta. Here, we take you through how melatonin helps the placental cells survive in a low-oxygen environment, a condition called hypoxia. In this environment, we found that melatonin helps the placental cells function and reduces their death rate by helping them recycle damaged parts to survive. Overall, our findings highlight melatonin as a powerful molecule playing a role in placental cells’ survival and how they function under stressful conditions. Since hypoxia is a common cause of pregnancy complications, melatonin could be a valuable aid for both animals and humans. Indeed, this study shows that melatonin is not just a sweet little pill; it is mighty and should only be used when truly needed.

## Introduction

Melatonin, mainly secreted by the pineal gland, is an amine hormone that plays a pivotal role in regulating reproductive physiology in mammals (Reiter *et al.* 2009). In this context, the effects of melatonin on the reproductive system and breeding management in sheep have long been explored (Palacin *et al.* 2011, Abecia *et al.* 2019). Melatonin is involved in the maternal recognition of pregnancy and in reducing oxidative stress, thus improving embryo quality (Forcada *et al.* 2006, Casao *et al.* 2010). Recently, much attention has been paid to its regulatory function for conceptus development in normal and compromised pregnancies (Vázquez *et al.* 2009, 2013, Casao *et al.* 2019). In our previous study, we reported that exogenous melatonin supplementation ameliorates uteroplacental interaction during the early stage of pregnancy, leading to an increased number of embryos per ewe (Viola *et al.* 2024). Furthermore, in sheep fed with a restricted diet, exogenous melatonin upturns embryo viability rate (Vázquez *et al.* 2013). Therefore, we surmise that the conceptus benefits from melatonin supplementation mainly when it is forced to grow in a suboptimal environment.

On the other hand, melatonin operates at the cellular level by scavenging reactive oxygen species (ROS), thus potentiating antioxidant defenses (Reiter *et al.* 2016). Melatonin reduces ROS levels in oocytes *in vitro* maturation and during blastocyst development in livestock species (Loren *et al.* 2017). This made melatonin a putative partner for trophoblast cells to overcome the early stage of pregnancy, which physiologically occurs under hypoxic conditions (Pringle *et al.* 2009, Bagchi & Bagchi 2024); however, how melatonin orchestrates cell adaptive response has not been clarified. In that context, an *in vitro* system allows studying cell mechanism dynamics that cannot be assessed in the whole organism.

Previous studies have reported that prolonged or severe hypoxia can alter trophoblast cell functionality in association with dysregulation of the autophagic response (Sagrillo-Fagundes *et al.* 2018, Carvajal *et al.* 2021). Autophagy is a highly conserved detoxifying mechanism involving the catabolism of damaged proteins and organelles that drives the maintenance of cellular growth, differentiation, and homeostasis in response to stressful conditions, including hypoxia (Redza-Dutordoir & Averill-Bates 2021). In human term-placental cells exposed to hypoxia/reoxygenation (H/R), melatonin suppresses pro-inflammatory response by inducing autophagy (Sagrillo-Fagundes *et al.* 2018), suggesting its ability to support placental homeostasis. However, melatonin-mediated autophagy differentially drives cell fate in placental cells derived from physiological or compromised pregnancies (Sagrillo-Fagundes *et al.* 2019). Together, these observations suggest the interconnected roles of hypoxia, autophagy, and melatonin in regulating trophoblast development and survival.

Therefore, this study is the first to explore the melatonin effect on early placenta cells’ behavior as well as its potential mitigating effect in CoCl_2_-induced hypoxia. Using previously characterized ovine primary trophoblast cells (Viola *et al.* 2023), we tested cell functionality, antioxidant activity, and autophagy cell fate modulation in a melatonin-enriched normal vs suboptimal environment.

## Methods

### Reagents

**Figure 1:**
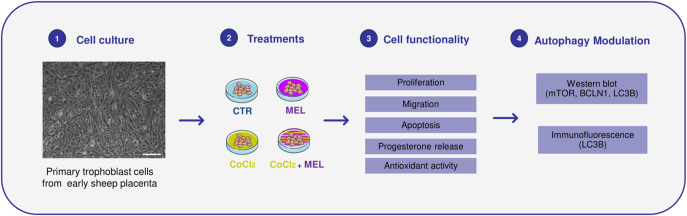
Experimental design. The flowchart shows the experimental trial setup. Cell culturing (step 1): ovine primary trophoblast cells (oTCs) previously isolated and characterized were cultured in a trophoblast growth medium at 37°C in an atmosphere of 5% CO2 until 80% confluence (Toschi *et al.* 2024). Treatments (step 2): cells were subjected to 24 h treatment with 250 μM melatonin (MEL), 200 μM CoCl_2_ to simulate a hypoxic environment (CoCl_2_), and 250 μM melatonin + 200 μM CoCl_2_ to study melatonin effect on stressed trophoblast cells. Cell functionality (step 3): the effect of previous treatments on trophoblast cells’ behavior was observed in proliferation, apoptosis, migration, progesterone release, and antioxidant activity. Autophagy modulation (step 4): Western blot analysis and immunofluorescence cell staining were performed to explore autophagy-related markers’ regulation through several stages of the autophagy process, including mTOR modulation, autophagy-related protein (BCLN1), and autophagosome protein detection (LC3B).

All chemicals, unless otherwise indicated, were obtained from Sigma Chemical Co. For cell isolation and culture, the following chemicals were used: Dulbecco’s Modified Eagle Medium, DMEM/F12 (Gibco, USA; 21331020); Minimal Essential Medium, MEM (M4655); penicillin-streptomycin (Gibco, 15140122); fetal bovine serum (FBS; Gibco, 10270106); l-glutamine (G7513); sodium pyruvate (S8636); non-essential amino acids (Gibco, 11140035); insulin (I9278); and dimethyl sulfoxide (DMSO; D2650), melatonin (M5250), cobalt chloride (CoCl_2_, 15862). For western blotting and immunocytochemistry analyses, the following antibodies were used: mouse anti-alpha-tubulin (aTUB, 1:10,000, T5168); rabbit anti-mTOR (mTOR, 1:1,000, Cell Signaling, USA; 2983); rabbit anti-pmTOR (pmTOR, 1:1,000, Cell Signaling, 5536); mouse anti-BCLN1 (BCLN1, 1:1,000, Santa Cruz Biotechnology, USA; sc-48341); rabbit anti-microtubule-associated protein light chain 3B (LC3B, 1:100 or 1:1,000, Invitrogen, USA; 46286); mouse anti-melatonin receptors (MEL-1A/B-R, 1:600, Santa Cruz Biotechnology, 398788); rabbit anti-glucose transporter-1 (GLUT1, 1:500, Novus Biologicals, USA; 120-1539); 4′,6-diamidino-2-phenylindole (DAPI; Thermo Scientific, USA); Alexa Fluor 488 goat anti-rabbit (1:500, Invitrogen, A11034); and horseradish peroxidase (HRP)-conjugated secondary antibodies (1:15,000, Immunopure goat anti-rabbit and anti-mouse IgG, Thermo Fisher Scientific); 5-bromo-2′-deoxyuridine (BrdU) (19160), anti-BrdU (1:100, B2531), DNase I (11284932001, Roche, Switzerland). The experimental design is shown in Fig. 1.

### Cell culture and treatments

**Figure 2:**
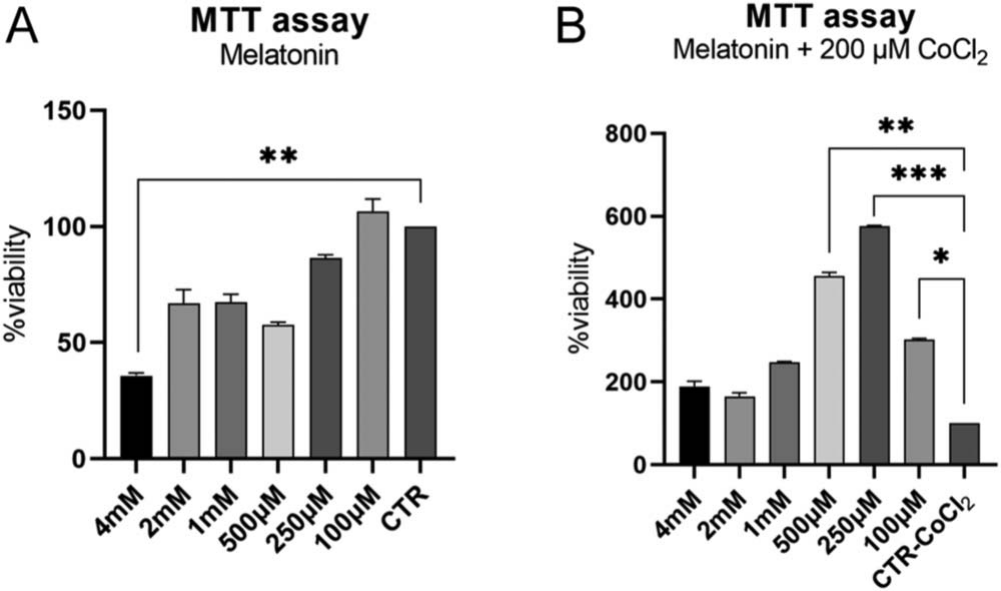
oTCs viability. MTT test was performed to assess oTCs viability in a dose-dependent manner for melatonin. (A) oTCs were 24 h exposed to different melatonin concentrations with or (B) without 200 μM CoCl_2_. Data are expressed as mean ± SEM (*P < 0.05; **P < 0.01; ***P < 0.001).

A previously characterized primary trophoblast cells from early sheep placenta (oTCs) was employed for *in vitro* analyses (Viola *et al.* 2023, 2024). Trophoblast cells were obtained from multiple placentas of multiparous ewes from day 21–23 of pregnancy. To reduce donor-to-donor variation and obtain a representative cell population, the samples were pooled before further processing. Cells were cultured in trophoblast growth medium defined as control (CTR), comprising DMEM-F12 supplemented with 100 UI/L penicillin and 100 μg/mL streptomycin, 10% FBS, 2 mM l-glutamine, 1 mM sodium pyruvate, 0.1 mM non-essential amino acids, and 4 μg/mL insulin. Cells were incubated at 37°C in an atmosphere of 5% CO2, and the culture medium was replaced every 48 h until 80% confluence. oTCs were starved for 6 h with MEM and then subjected to the following 24 h treatments. Melatonin concentration was established based on a previous dose-dependent assay performed with both single and combined treatments (Fig. 2): 250 μM melatonin (MEL), 200 μM CoCl_2_, and 250 μM melatonin + 200 μM CoCl_2_ (CoCl_2_ + MEL). oTCs were used between passages 2 and 8.

### MTT assay

In total, 3,000 oTCs were seeded in a 96-well plate and maintained in the normal trophoblast growth medium until 70% confluence. After 6 h of starvation, cells were treated for 24 h with melatonin (4, 2, 1 mM, 500, 250, 100 μM) with or without 200 μM CoCl_2_. Then, cell viability was assessed using the CyQUANT MTT Cell Proliferation Assay Kit (Invitrogen, V13154) as reported by the manufacturer’s procedure. oTC viability was evaluated by reading absorbance at 570 nm, including 630 nm as a reference (Bio-Rad Model 680, microplate reader, USA). The MTT assay was repeated in quadruplicate for each treatment three different times.

### Proliferation assay

The BrdU incorporation assay was performed to assess cell proliferation. For this, 25,000 cells were seeded on a four-well chamber slide in duplicate (Nunc Lab-Tek II Chamber Slide System). Once 70% confluence was reached, oTCs were starved for 6 h and then treated for 24 h according to the above-mentioned treatment and incubated with 10 μM BrdU. Cells were fixed in 4% formaldehyde for 15 min, permeabilized at RT with 0.1% Triton X-100 for 15 min, and then treated with 2 M HCl at RT for 30 min; blocking was performed using 1% BSA at RT for 1 h, and anti-BrdU was added and incubated overnight at 4°C. Subsequently, the cells were incubated at RT for 1 h with the secondary antibody Alexa Fluor 488 goat anti-mouse and counterstained with DAPI for 10 min at RT. Between every step, the cells were washed three times with PBS at RT for 2 min. The number of proliferative cells was calculated for each group by calculating the ratio between the number of BrdU-positive cells and the total number of nuclei. At least 1000 nuclei were analyzed for each treatment by randomly selecting fields. Images were acquired with a Leica AF6000 LX fluorescent microscope (Leica Microsystems, Germany) equipped with a Leica DFC350FX digital camera controlled by LAS AF software (Leica Microsystems).

### Apoptosis assay

For this, 60% confluent oTCs were cultured according to the treatments on a four-well chamber slide in duplicate. ApopTag® Fluorescein *in situ* Apoptosis Detection Kit (S7110 Millipore, France) was used to assess apoptotic cell numbers following the manufacturer’s protocol, including a negative control. Meanwhile, a positive control was included by pre-treating cells with 5 μg/mL DNase I for 60 min at 37°C. At least, 400 cells for each treatment were counted, and the positive cells/total cells ratio was calculated as apoptosis %. Pictures were captured with a Leica SP8 confocal fluorescent microscope (Leica Microsystems) at 20X magnification.

### Migration assay

oTC migration was conducted using 24-well Transwell plates, following the procedure described by Pijuan *et al.* (2019). For this, 50,000 cells in 100 μL of serum- and insulin-free DMEM were seeded into the upper chamber of an 8 μm Transwell chamber (Costar, 3422, USA), to which 600 μL of growth medium was added, according to the treatment, into the lower chamber. After 12 h of culture, the cells on the upper side of the inserts were removed and washed twice with PBS. The inserts were fixed in 4% formaldehyde for 15 min to evaluate migration onto the lower surface. The dried chamber was stained with DAPI for 10 min at RT and washed with PBS. At least 5,000 cells were counted in ten random non-overlapping fields acquired at a magnification of 10-X with a Leica AF6000 LX (Leica Microsystems) fluorescent microscope. The percentage of migrated cells was calculated as the ratio between the number of migrated cells and total cells.

### Progesterone release

Progesterone extraction was performed following the DetectX® Steroid Liquid Sample Extraction Protocol provided by Arbor Assays, with slight modifications. Briefly, oTC culture medium was collected, centrifuged for 15 min at 13,520 ***g*** to discard cellular debris, and the suspension was stored at −80°C until extraction. 400 μL was processed with 2 mL of diethyl ether in a glass tube, vortexed for 1 min, left at RT for 5 min, and kept for 1 h at −80°C. The procedure was repeated twice. Then, the ether was transferred to a glass vial for evaporation by ultracentrifugation in a SpeedVac for 3 h (Thermo Savant, USA; SC110A-115 SpeedVac Plus Concentrator) and stored at −20°C. Progesterone concentration was assessed with an enzyme-linked immunosorbent assay kit (Progesterone ELISA, DRG Diagnostics GmbH, Germany). Analyses were repeated twice on at least five different endpoint analyses. The progesterone concentration was expressed as pg/100,000 cells.

### Immunofluorescence

oTCs were cultured on four-well chamber slides (Nunc Lab-Tek II Chamber Slide System) under different treatment conditions. Cells were fixed with 4% paraformaldehyde at RT for 10 min, washed twice with Tris-buffered saline (TBS) for 3 min each, and then non-specific antigen sites were blocked with 1% BSA at RT for 2 h. oTCs were incubated at 4°C overnight with LC3B antibody. After washing twice with TBS, the secondary antibody Alexa Fluor 488 goat anti-rabbit was incubated at RT for 1 h in the darkness, and nuclei were stained with DAPI for 10 min. Meanwhile, the no-primary-antibody control and negative control were processed similarly; analysis was performed twice on two different endpoints. A Leica SP8 confocal fluorescent microscope (Leica Microsystems) was used to capture pictures at 20X and 63X.

### Antioxidant activity

Hydrogen peroxide (H_2_O_2_) was measured in oTC medium. For this, 3,000 oTCs were seeded in a 96-well plate and maintained in the normal trophoblast growth medium. Once 70% confluence was reached, oTCs were exposed to previously reported culture conditions and treated as described by the ROS-Glo H_2_O_2_ Assay Kit (Promega, USA; G8220). Eight replicates were performed for each treatment. The plate was read with Victor 3 (PerkinElmer, USA).

### Western blot

Protein extraction was performed from 90% confluent oTCs on six-well plates under the indicated experimental conditions. Cells were washed with ice-cold PBS and then lysed for 10 min on ice in 200 μL lysis solution composed of 10 mM Tris-HCl pH 7.4, 150 mM NaCl, 1 mM EDTA, 1% Triton X-100, 0.1% SDS, 0.5% sodium deoxycholate, and 0.01% sodium azide, a protease inhibitor cocktail (1:100), 1 mM sodium orthovanadate, and 1 mM phenylmethylsulfonyl fluoride. All lysates were centrifuged at 4°C for 15 min at 15,000 *g*, and the amount of protein in the supernatants was quantified by DC Protein Assays (Bio-Rad Laboratories, USA) following the protocol’s instructions. 10 μg of total protein was resolved on 7.5% or 15% polyacrylamide gels and transferred to 0.2 μm nitrocellulose (mTOR, pmTOR, BCLN1, GLUT1, MEL-1A/B-R, aTUB) or polyvinylidene difluoride (LC3B, aTUB) blotting membranes (Amersham Protran Premium) overnight. Membranes were blocked at RT for 1 h in 10% BSA TBS (TBS-Tween, 10 mM Tris, and 150 mM NaCl, pH 7.4, 0.1% Tween 20), then incubated overnight at 4°C with the primary antibodies. Membranes were washed for 30 min in TBS-Tween and incubated at RT for 1 h with HRP-conjugated secondary antibody. Negative controls were processed similarly. The membranes were washed in TBS–Tween and incubated for 5 min at RT with Clarity Western ECL Substrate (Bio-Rad Laboratories). Analyses were repeated twice on two different endpoints. The proteins were visualized by exposing the membranes to autoradiographic CL-XPosure Film (Thermo Fisher Scientific). Western blotting results were acquired with an EPSON Perfection V39 scanner. Densitometry analysis was performed using ImageJ Fiji 1.53 s (National Institutes of Health, USA; https://imagej.nih.gov/ij/), repeating each band reading three times. The relative protein expression was calculated using aTUB as a reference control.

### Gene analysis

The procedure followed the protocol described in Viola *et al.* (2024). Gene expression analysis was performed on glucose transporter 1 (*GLUT1*, U89029, 5′-CCA​TTG​CTG​TTG​CCG​GTT​T-3′; 3′-TGG​AAG​CAC​ATG​CCC​ACA​A-5′) and glucose transporter 3 (*GLUT3*, NM_001009770 5′-AGG​CGC​AAC​TCA​ATG​CTT​ATT-3′, 3′-TGC​AGA​ATC​CCA​TAA​GGC​AGC-5′). RT-qPCR was performed on five independent replicates.

### Statistical analysis

Cell proliferation, apoptosis, and migration data were analyzed using Fisher’s Exact Test, whereas antioxidant activity and densitometry data were analyzed using the Kruskal–Wallis one-way analysis of variance by ranks. When multiple comparisons were significant, pairwise comparisons were performed using the Mann–Whitney U test. MTT data were analyzed using one-way ANOVA followed by Dunnett’s multiple comparison test (Ebegboni *et al.* 2019). Unless otherwise indicated, each *in vitro* assay was conducted with at least three independent replicates. Data are presented as mean ± SEM (standard error of the mean). All statistical analyses were performed using GraphPad Prism 9 software. Asterisks in the graphs indicate statistically significant differences: *P < 0.05, **P < 0.01, ***P < 0.001, ****P < 0.0001.

## Results

### MTT assay in the oTCs model

MTT assay was assessed to determine cell viability under melatonin and CoCl_2_ treatments (Fig. 2). Following only melatonin treatment, oTCs showed a significant decrease in viability when cells were subjected to 4 mM melatonin (*P* < 0.01). Despite no statistical differences, cell viability was enhanced with 100 μM melatonin. Then, melatonin’s ability to support oTCs viability was evaluated under hypoxic culture conditions. In that condition, cell viability strongly increased when cells were exposed to 100, 250, and 500 μM melatonin (*P* < 0.05; *P* < 0.01; *P* < 0.001); whereas no effect was detected under 1, 2, and 4 mM melatonin. 250 μM melatonin did not affect cell viability in normal conditions; meanwhile, it positively supported oTCs survival under hypoxic conditions (*P* < 0.001). Considering these results, oTCs were subjected to 250 μM melatonin in the following functional tests.

### MEL-1A/B-R, GLUT1 and GLUT3 expression

**Figure 3:**
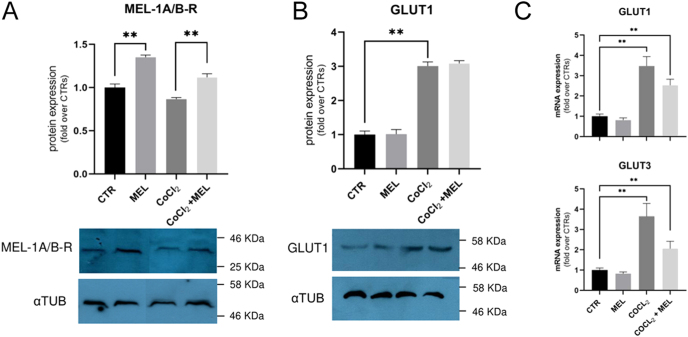
Melatonin receptors and glucose transporters’ expression in oTCs. (A) Immunoblot showed increased melatonin receptors’ expression when cells were exposed to 250 μM melatonin. (B) A hypoxic environment stimulated GLUT1 expression compared to normal culture conditions, even in the presence of melatonin supplementation. aTUB was used as a reference protein. (C) *GLUT1* and *GLUT3* mRNA expression was up-regulated in CoCl_2_ environment. Densitometry and gene expression analysis show data as mean ± SEM (**P < 0.01).

Western blot analysis showed that in melatonin-treated oTCs, an increase of melatonin receptors was observed both in hypoxic and normal conditions (*P* < 0.01) (Fig. 3A). Furthermore, GLUT1 expression was markedly increased following CoCl_2_ treatment compared to the non-hypoxia-induced environment (Fig. 3B). *GLUT1* and *GLUT3* upregulation was also confirmed in mRNA expression of CoCl_2_-treated cells compared to those grown in non-hypoxic conditions.

### Proliferation rate, migration activity, and progesterone release

**Figure 4:**
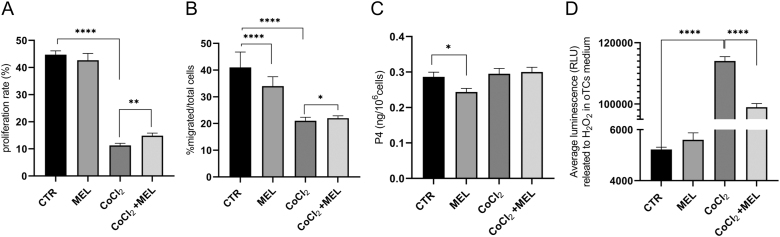
Functional activity in melatonin-treated oTCs in normal vs hypoxic environments. (A) The graph reports the proliferation rate expressed as a percentage of the total cells. Melatonin increased proliferative activity only following CoCl_2_ treatment, whereas it did not impact mitogen activity in normal trophoblast growth medium compared to CTR. (B) The migrative activity was improved by melatonin only in a hypoxic environment, whereas in normal culture conditions, melatonin reduced oTCs activity. Data were reported as a ratio of migrated cells to the total. (C) A slight reduction in P4 release was observed in MEL oTCs, whereas the hypoxia-induced environment did not affect hormone secretion. (D) The effect of melatonin treatment on reactive oxygen species metabolism was tested in oTCs. In normal growth conditions, melatonin did not impact trophoblast cells’ response; nevertheless, melatonin led to a significant reduction in H_2_O_2_ when cells were exposed to a hypoxic culture environment. Data are reported as mean ± SEM (*P < 0.05; **P < 0.01; ****P < 0.0001).

First, the BrdU assay displayed that melatonin did not improve oTCs proliferation in normal growth medium (Fig. 4A). CoCl_2_ drastically decreased the mitogen index compared to CTR (*P* < 0.0001). Still, melatonin restored proliferative activity in CoCl_2_-treated oTCs (*P* < 0.01). Melatonin reduced migratory activity compared to normal culture conditions (Fig. 4B, *P* < 0.0001). Migration was slower in CoCl_2_-treated oTCs than in the CTR (*P* < 0.0001); additionally, melatonin supplementation enhanced oTCs migration in hypoxic conditions. Finally, progesterone (P4) release in the culture medium was not affected by CoCl_2_ exposure, but melatonin reduced hormonal secretion in normal trophoblast growth medium (Fig. 4C, *P* < 0.05).

### Antioxidant activity

As illustrated, in the CoCl_2_ environment, oTCs struggled to minimize H_2_O_2_ levels compared to CTR (Fig. 4D, *P* < 0.0001). No effect was observed in melatonin-treated oTCs in normal trophoblast medium; however, a significant decrease in H_2_O_2_ was detected when CoCl_2_-treated oTCs were simultaneously exposed to melatonin (*P* < 0.0001).

### Autophagy and apoptosis in melatonin- and CoCl_2_-treated trophoblast cells

**Figure 5:**
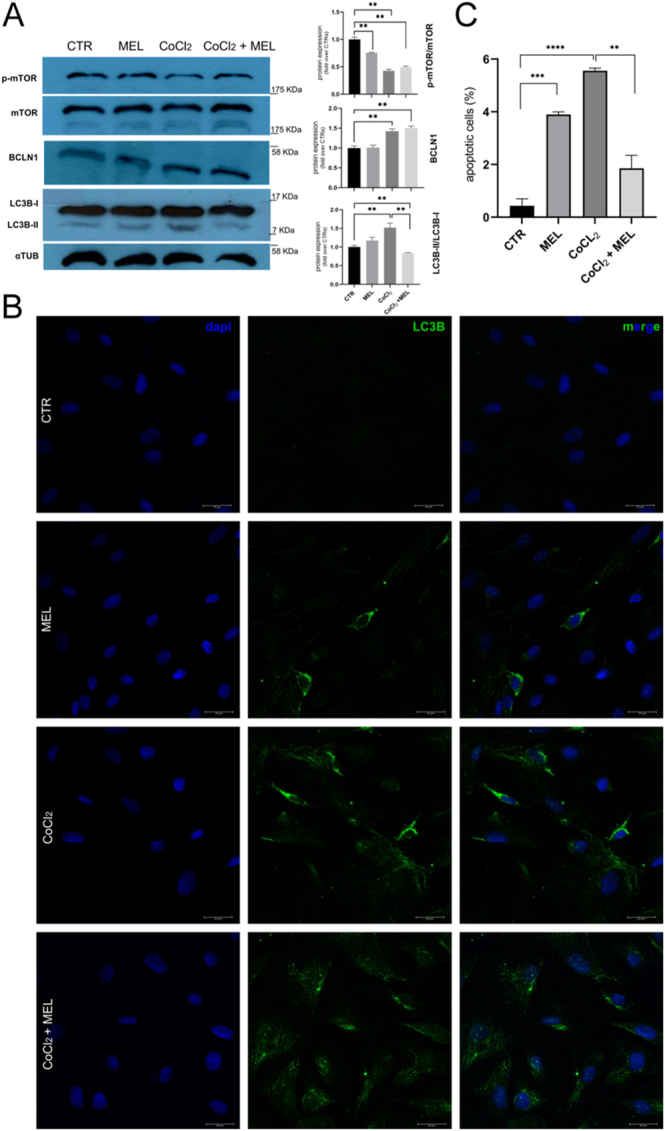
Autophagy markers’ modulation and apoptosis in sheep trophoblast cells. (A) Western blot showed the main autophagy markers’ profile in oTCs subjected to melatonin in or not hypoxic environment. mTOR suppression was observed in melatonin and CoCl_2_ cells, either separately or in co-exposure conditions. BCLN1 expression was enhanced only when cells grew in a hypoxic environment. The increased LC3BII/LC3BI ratio suggested that the autophagy process was finalized only in CoCl_2_-treated oTCs, whereas LC3BII expression diminished in CoCl_2_-MEL cells. (B) Autophagosome marker was detected by immunofluorescence cell staining after 24 h of melatonin and CoCl_2_ treatment. LC3B-positive cells were observed in both melatonin and hypoxic environments, whereas they were not in normal environments. LC3B signal was more accumulated in cells exposed to CoCl_2_ alone compared to those concomitantly treated with melatonin, suggesting a potential alteration in autophagic flux. Please note that immunofluorescence images are provided for qualitative purposes only to illustrate the intracellular distribution pattern of LC3B signal and are not intended for quantitative analysis (nuclei staining, blue; LC3B, green; scale bar: 25 μm). (C) TUNEL assay showed an increase in cell death in both melatonin and CoCl_2_-treated oTCs compared to CTR; meanwhile, melatonin decreased the apoptosis rate in hypoxic growth conditions. Data are expressed as mean ± SEM and %, respectively (**P < 0.01; ***P < 0.001; ****P < 0.0001).

Autophagy markers’ expression was evaluated according to the different treatments (Fig. 5). mTOR phosphorylation decreased in all treatments compared to CTR (*P* < 0.01), but its expression remained unaltered following melatonin supplementation under hypoxic conditions (Fig. 5A). BCLN1 was overexpressed in both hypoxia conditions compared to CTR. The increase of BCLN1 and LC3BII/LC3BI ratio in CoCl_2_ oTCs suggested an increased autophagic activity (*P* < 0.01), as also evidenced by LC3B detection in immunofluorescence staining (Fig. 5B). LC3B was also observed in melatonin supplementation; however, autophagic protein expression did not differ compared to CTR (Fig. 5B). LC3B was detected in concomitant treatment with CoCl_2_ and melatonin, but the LC3BII/LC3BI ratio was decreased both compared to normal and CoCl_2_ oTCs (*P* < 0.01). Finally, the TUNEL assay (Fig. 5C) showed increased apoptotic activity in both melatonin- and CoCl_2_-exposed cells compared to normal growth conditions (*P* < 0.001; *P* < 0.0001); meanwhile, a decrease in cell death was observed when CoCl_2_ oTCs were simultaneously subjected to melatonin (*P* < 0.01).

## Discussion

The present study explored the effects of melatonin on trophoblast cells’ response to a suboptimal environment during the early stage of pregnancy in sheep. Here, we provide evidence that melatonin exerts a cytoprotective effect in hypoxia-inducing environments, preserving trophoblast cell function by attenuating oxidative stress and modulating the apoptosis–autophagy balance toward an adaptive response. Melatonin fundamentally augments the placental adaptive strategy, acting as a mitigating agent when trophoblast cells are subjected to hypoxic conditions, as opposed to normoxia. Since placental response research requires the use of a dynamic system, oTCs *in vitro* model was used to study how melatonin exposure triggers trophoblast adaptive capacity. So, we tested the hypothesis that melatonin orchestrates the functionality and cell fate decisions of trophoblast cells under a hypoxic environment, which physiologically occurs during the early stage of pregnancy.

### Melatonin receptors and glucose transporters’ expression

It is currently unknown what the real melatonin concentration is that the ovine placenta is physiologically exposed to during initial placentation. The range of melatonin used in previous *in vitro* studies notably varies among species and cellular models. Sheep oocytes were exposed to much lower concentrations (up to 1 pM melatonin, Tian *et al.* 2017) than bovine embryos (10 mM, Papis *et al.* 2007) or human primary trophoblast cells (1 mM, Sagrillo-Fagundes *et al.* 2016). To determine the optimal melatonin concentration for our system, we conducted a dose-dependent viability assay, both with and without CoCl_2_. Melatonin *per se* did not significantly impact cell viability up to higher concentrations (4 mM); however, following CoCl_2_ co-exposure, 250 μM melatonin was the most robust support for oTCs survival, and thus was selected for further experiments.

Moreover, to confirm the effectiveness of those treatments in our system, we assessed MEL-1A/B-R, GLUT1, and GLUT3 expression. As expected, melatonin supplementation, regardless of the presence of CoCl_2_, leads to an increased expression of melatonin receptors in oTCs. Upregulation of melatonin receptors by *in vitro* melatonin treatment has previously been reported in porcine granulosa cells (He *et al.* 2016), bovine theca cells (Wang *et al.* 2019), as well as in sheep placenta exposed to melatonin during the peri-implantation period (Viola *et al.* 2024).

Thereafter, CoCl_2_ supplementation induced *GLUT1* mRNA and protein expression, suggesting that the treatment activated hypoxia-inducible gene as previously reported in trophoblast-derived human BeWo and rat Rcho-1 cell lines (Hayashi *et al.* 2004), and trophoblast cells isolated from normal term placenta (Esterman *et al.* 1997) in response to oxygen deficiency. Increased *GLUT3* mRNA expression confirmed the onset of hypoxia-related molecular machinery, as reported in IUGR human placenta associated with ischemic hypoxia (Janzen *et al.* 2013), as well as in BeWo cells cultured in a prolonged hypoxic environment (Baumann *et al.* 2007). Our data confirm that the induction of hypoxic conditions in placental cells leads to increased expression of *GLUT1* and *GLUT3* as an adaptive metabolic response that enhances glucose uptake during periods of oxygen shortage.

### Proliferation and migration activity

Regarding melatonin’s effect on cellular functionality, contradictory data emerge from the literature; that is due to the different cell types (primary cells vs cell lines), origin (cancer vs normal cells), and applied dose and time of melatonin (Lanoix *et al.* 2012, 2013, Sagrillo-Fagundes *et al.* 2019). Here, we observed that 250 μM melatonin did not affect sheep trophoblast cell proliferation, while migration activity was reduced compared to normal conditions (Fig. 4A and B). In other studies, *in vitro* supplementation of 1 mM melatonin had an inhibitory effect on cell proliferation (Cui *et al.* 2006) and invasion/migration (Alvarez-García *et al.* 2013) on human umbilical vein endothelial cells. On the contrary, a lesser melatonin concentration leads to increased mitogen activity in porcine early trophoblast cells subjected to 50 μM melatonin (Bae *et al.* 2020). More recently, Yang *et al.* (2025) found that 10 μM melatonin treatment strongly promotes HTR-8/SVneo cell migration and proliferation. Therefore, melatonin’s influence in controlling cell proliferation and migration seems to be strictly dependent on its concentration.

### Antioxidant activity

Otherwise, evidence on which all *in vitro* experiments converge is the ability of melatonin to mitigate oxidative stress (Sagrillo-Fagundes *et al.* 2018, Fu *et al.* 2022, Zhai *et al.* 2023, Yang *et al.* 2025). In our experiments, exposure to hypoxic conditions strongly reduces cell proliferation and migration, and increases H_2_O_2_ levels; however, concomitant melatonin supplementation has a rescue effect, as supported by our data regarding mitogen, migrative, and antioxidant capacity (Fig. 4).

These results are consistent with previous studies showing that melatonin supplementation ameliorated the H_2_O_2_-induced reduction in porcine trophoblast cell viability and significantly decreased ROS production (Fu *et al.* 2022). Other results obtained from human primary villous trophoblast cells indicate that exogenous melatonin treatment may afford protection against H/R-induced damage, thus enhancing placental cell survival (Sagrillo-Fagundes *et al.* 2018). Moreover, in the human trophoblast cell line (HTR-8/SVneo cells), melatonin supplementation significantly downregulated cellular ROS levels in an H_2_O_2_-induced oxidative stress model (Yang *et al.* 2025). In this view, melatonin is considered a potent radical scavenger, performing its protective action during pregnancy (Reiter *et al.* 2024). Our study also confirmed the ability of melatonin to support antioxidant activity in a hypoxic environment, as demonstrated by the fact that CoCl_2_-treated cells better metabolize H_2_O_2_ only following melatonin supplementation.

### Progesterone release

Another interesting aspect related to melatonin treatment is the ability to regulate steroidogenesis. Several research studies have confirmed melatonin’s effect on progesterone release in humans (Webley & Leidenberger 1986, Webley *et al.* 1988), cattle (Webley & Luck 1986), and sheep (Durotoye *et al.* 1997). Melatonin enhanced P4 synthesis activity in ovine granulosa cells (Baratta & Tamanini 1992). Similarly, in our previous *in vivo* study, an increase in plasmatic P4 was observed in melatonin-treated ewes on day 21 of pregnancy (Viola *et al.* 2024), although the origin (placenta/pineal gland) of its secretion remains unclear. In this view, we assessed the P4 release in ovine trophoblast cells, and unexpectedly, it was reduced following melatonin supplementation. We suspect that the difference depends on treatment duration; oTCs were exposed to melatonin for 24 h, considered a shorter time than the 72 h commonly used in other human *in vitro* studies (Lanoix *et al.* 2013, Sagrillo-Fagundes *et al.* 2019). Based on this outcome, further experiments should consider prolonged melatonin treatment to assess the effect on trophoblast hormonal secretion in sheep.

### Autophagy and apoptosis balance

According to the fact that hypoxia is one of the main switch-on factors for autophagy (Li *et al.* 2015), we detected an increased protein expression of autophagic markers (BCLN1 and LC3BII/LC3BI ratio) in concomitance with a decreased phosphorylation of mTOR. These results were also supported by immunofluorescence imaging, evidencing an autophagosome accumulation in CoCl_2_-treated cells compared to CTR. Similarly, in humans, the expression of LC3 and BCLN1, as well as autophagosome formation, was significantly increased in early-onset preeclamptic placentas and in HTR8/SVneo cells following induction of oxidative stress (Gao *et al.* 2015). Interestingly, the supplementation of melatonin in an environment already compromised by CoCl_2_ did not influence mTOR phosphorylation and BCLN1 levels; however, a reduced rate of LC3BII conversion was detected in cells concomitantly treated with CoCl_2_ and melatonin. Immunofluorescence analysis of LC3B confirmed that *in vitro* induction of hypoxia triggers autophagy hyperactivation. Compared to CTR, both groups of CoCl_2_-treated cells displayed a higher incidence of positive cells; however, simultaneous supplementation of melatonin seems to attenuate the cytoplasmic engulfment of LC3B puncta, suggesting a more efficient use of the autophagic strategy.

Given that excessive autophagy can lead the cells toward a programmed cell death fate, we also decided to explore the rate of apoptotic cells in our experimental groups. Although the general incidence of apoptosis was very weak (overall average of 2.9%), following CoCl_2_ treatment, we recorded a higher percentage of apoptotic cells compared to other experimental conditions. Concomitant melatonin supplementation significantly reduces the incidence of apoptosis, consistent with optimized autophagy, although the levels remain lower than those of the control group.

Similarly, melatonin supplementation ameliorated the H_2_O_2_-induced reduction in porcine trophoblast cell viability and significantly decreased apoptosis (Fu *et al.* 2022). Sagrillo‐Fagundes *et al.* (2018) reported that melatonin acts as a cytoprotective molecule, able to protect against increased levels of apoptosis induced by H/R in primary human villous trophoblasts. Interestingly, under H/R conditions, cancer-derived cells such as BeWo enhanced autophagic activity to protect cells against apoptosis. Melatonin treatment blocks the rise in autophagy in BeWo cells, thereby contributing to their apoptosis. Conversely, in primary cytotrophoblast cells, H/R also enhances autophagic activity, which is further increased by melatonin, thereby contributing to cell survival (Sagrillo-Fagundes *et al.* 2019). Interestingly, Shen *et al.* (2018) postulated that in mouse granulosa cells, melatonin-mediated protection from oxidative damage passes through autophagy suppression rather than antioxidation.

Differently, when cells were subjected only to melatonin, although the levels of autophagic proteins such as BCLN1 and LC3BII remained unaltered, we observed an increased phosphorylation of mTOR associated with a higher incidence of LC3B-positive cells compared to CTR. This suggests that following melatonin supplementation, a small portion of cells activate autophagy. However, in the same conditions, we observed a significant increase in TUNEL-positive cells, suggesting that most of them undergo apoptotic cell death. According to the MTT results, this can be attributed to the high concentration of melatonin used for these experiments, which is effective in counteracting the hypoxic damage caused by CoCl_2_, but could be cytotoxic in normal culture conditions. This could also explain the decreased levels of progesterone release in melatonin-treated cells. In rabbit fibroblasts, melatonin treatment attenuated mTOR phosphorylation and increased the LC3BII-I ratio, suggesting an enhancement of the autophagic flux (Dong *et al.* 2024). Furthermore, it has been described that melatonin promotes mTOR-mediated autophagy in ovine luteal cells (Duan *et al.* 2024).

### Limitations of the study

Although the study employs primary cells, which offer a more physiologically *in vitro* model compared to immortalized cell lines, several limitations should be acknowledged. First, the absence of functional data across multiple time points and concentrations limits a comprehensive understanding of the dose-dependent effects of melatonin, particularly in terms of the kinetics and potential thresholds of melatonin’s actions under hypoxic conditions. Second, a limitation lies in the use of chemically induced hypoxia, which, although widely adopted, does not fully mimic the dynamics of oxygen deprivation. Third, while the study applied a 24 h hypoxic exposure, it would be valuable to explore the effects of prolonged treatments. Longer exposure periods may reveal potential compensatory mechanisms activated by melatonin over time and help determine whether some of the negative effects observed (i.e. reduced cell migration and decreased progesterone secretion) could be reversed or mitigated under sustained treatment conditions. Overall, while these limitations do not diminish the significance of the findings, they suggest directions for future studies that could further clarify the complexity of melatonin’s role under hypoxic conditions.

In conclusion, the present research is the first to focus on the effects of melatonin on *in vitro* trophoblast adaptive response in sheep. This study emphasizes the role of melatonin in supporting trophoblast functionality under hypoxic conditions, thereby aiding in the restoration of cellular homeostasis. In summary, melatonin influences cell fate depending on the culture environment. In normal conditions, exogenous melatonin could negatively affect cell functionality; however, its supplementation becomes strategic when trophoblast cells encounter unfavorable conditions such as hypoxia or starvation. This makes melatonin a promising compound for alleviating pregnancy disorders associated with placental insufficiency, such as preeclampsia and intrauterine growth restriction, both of which are characterized by reduced systemic melatonin levels and impaired autophagy activation (Zeng *et al.* 2016, Fantasia *et al.* 2022, Singh *et al.* 2024). Beyond its well-established antioxidant properties, which *per se* benefit in multiple pathological conditions, melatonin may confer additional advantages in gestational conditions involving impaired placental vascular function, which compromises both nutrient and oxygen delivery to the fetus. Such effects could be particularly relevant not only in compromised singleton, but also in multiple pregnancies, where placental capacity may be insufficient for all fetuses (Flinn *et al.* 2020). Furthermore, in both women and livestock species experiencing repeated early pregnancy losses (i.e. sheep with repeated return to estrus after first mating), melatonin supplementation could be especially advantageous, as the early stages of gestation involve a developing placental vascular network that, if inadequate, increases the risk of hypoxia-induced miscarriage. Nevertheless, the exact mechanisms by which melatonin modulates hypoxia-related pathways, including apoptosis and autophagy, remain to be fully elucidated, and further research is needed to define optimal timing and dosing for therapeutic application.

## Declaration of interest

The authors declare that there is no conflict of interest that could be perceived as prejudicing the impartiality of the work reported.

## Funding

The Department of Veterinary Sciences, Università degli Studi di Torino, partially covered the open access APC. This work has also been supported by the UNITA Alliance and the Erasmus + funding.

## Author contribution statement

IV and PT conceived and planned the study. IV, PA, IM, EQ, and PT performed the laboratory analyses. IV, FC, SM, JAA, and PT performed data analysis and interpretation. IV, JAA, and PT conducted a literature search and wrote the paper. PT supervised the project. All authors reviewed the results and approved the final version of the manuscript.
